# Characterization and Cytotoxicity Assessment of the Lipophilic Fractions of Different Morphological Parts of *Acacia dealbata*

**DOI:** 10.3390/ijms21051814

**Published:** 2020-03-06

**Authors:** Cátia S. D. Oliveira, Patrícia Moreira, Judite Resende, Maria T. Cruz, Cláudia M. F. Pereira, Artur M. S. Silva, Sónia A. O. Santos, Armando J. D. Silvestre

**Affiliations:** 1 CICECO—Aveiro Institute of Materials, University of Aveiro, 3810-193 Aveiro, Portugal; cs.oliveira@ua.pt (C.S.D.O.); judite.resende@ua.pt (J.R.); santos.sonia@ua.pt (S.A.O.S.); 2 CNC—Center for Neuroscience and Cellular Biology, University of Coimbra, 3004-504 Coimbra, Portugal; patriciaraquel_jm@hotmail.com (P.M.); trosete@ff.uc.pt (M.T.C.); claudia.mf.pereira@gmail.com (C.M.F.P.); 3 LAQV-REQUIMTE, Department of Chemistry, University of Aveiro, 3810-193 Aveiro, Portugal; artur.silva@ua.pt; 4 Faculty of Pharmacy, University of Coimbra, 3000-548 Coimbra, Portugal; 5 Faculty of Medicine, University of Coimbra, 3000-548 Coimbra, Portugal

**Keywords:** *Acacia dealbata*, forest biomass, biorefinery, lipophilic compounds, GC–MS analysis, cytotoxicity, nutraceutical applications, pharmaceutical applications

## Abstract

*Acacia dealbata* biomass, either from forest exploitation or from the management of invasive species, can be a strategic topic, namely as a source of high-value compounds. In this sense, the present study aimed at the detailed characterization of the lipophilic components of different morphological parts of *A. dealbata* and the evaluation of their cytotoxicity in cells representative of different mammals’ tissues. The chemical composition of lipophilic extracts from *A. dealbata* bark, wood and leaves was evaluated using gas chromatography-mass spectrometry (GC–MS). Terpenic compounds (representing 50.2%–68.4% of the total bark and leaves extracts, respectively) and sterols (60.5% of the total wood extract) were the main components of these extracts. Other constituents, such as fatty acids, long-chain aliphatic alcohols, monoglycerides, and aromatic compounds were also detected in the studied extracts. All the extracts showed low or no cytotoxicity in the different cells tested, demonstrating their safety profile and highlighting their potential to be used in nutraceutical or pharmaceutical applications. This study is therefore an important contribution to the valorization of *A. dealbata*, demonstrating the potential of this species as a source of high value lipophilic compounds.

## 1. Introduction

*Acacia dealbata* (silver wattle or mimosa) is a woody legume, introduced in Europe in the 18th century, which then invaded Atlantic and Mediterranean climates, from Portugal to Italy [1,2,3,4]. Soil disturbances, fires, and climate changes, leading to increasing areas susceptible to colonization [3] have probably contributed to *A. dealbata* invasion of many regions. Although this species produces wood suitable for good quality pulp fibers [5,6,7], its industrial applications have been mainly focused on wood for furniture [8], gum, or as a substitute of gum arabic, on bark for tanning production [7,9,10,11], and on flowers for absolute oil production, and in the flavor and perfumes industry [12]. In addition, *A. dealbata* has been traditionally used to control soil erosion, and as an ornamental species [13].

Notwithstanding, the invading behavior of *A. dealbata* turned it into a major concern for the forestry sector, particularly for the pulp and paper industry and the respective *Eucalyptus* species plantations [14]. Actually, *A. dealbata* has been considered a residue for pulp industry, being left in the forest or used essentially an energy source [9,15,16]. Thus, the valorization of this species for high value applications is of major importance for countries where pulp and paper industry has a high impact. In fact, this industry is increasingly on the alert for waste reduction and recovery of residual forest biomass.

So far, only a few studies have been focused on searching innovative ways to add value to *A. dealbata*, demonstrating that it is particularly interesting as a source of high-value compounds [8,15,17]. Nevertheless, these studies have only focused on specific families [8,15], specific tree parts [17], or even neglected the quantification of the compounds detected [8]. In fact, while different phytosterols were identified and quantified in *A. dealbata* wood and bark [15], only two terpenic compounds were reported as constituents of this species, namely lupenone in leaves, flowers and seeds and lupeol in leaves [8], and in both cases without quantitative data. Some fatty acids, long-chain aliphatic alcohols, and monoglycerides were also identified in *A. dealbata* bark [17], but their presence in other parts of the tree as well as their abundance remain unknown. 

Additionally, the cytotoxicity of *A. dealbata* lipophilic extracts has not been exploited so far, although a wide range of biological properties have been already reported for some of its constituents, such as the anti-inflammatory, antidiabetic, antiviral, and anticancer activities of lupenone [18] and anti-inflammatory, antiproliferative, and anticarcinogenic activities associated to spinasterol and 22,23-dihydrospinasterol [19,20,21,22], which were identified as the major sterols in *A. dealbata* bark and wood [15].

In this vein, the detailed knowledge about the lipophilic composition of different morphological parts of *A. dealbata,* envisaging their integrated exploitation, remains scarce, particularly concerning all the important families of lipophilic compounds, like fatty acids, long-chain aliphatic alcohols, sterols, terpenic compounds, or monoglycerides. 

In order to fill this gap, this work aims at a systematic study concerning the lipophilic composition of the bark, wood, and leaves of *A. dealbata*, by gas chromatography-mass spectrometry analysis (GC–MS). Additionally, and in order to evaluate the potential exploitation of *A. dealbata* lipophilic fractions for pharmaceutical (in oral or topical applications) and nutraceutical purposes, the cytotoxicity in different cell lines representative of different tissues and organs, such as the brain, innate immune system, skin, lung, and liver is also disclosed.

## 2. Results and Discussion

### 2.1. Lipophilic Extractives Yield of A. dealbata Bark, Wood and Leaves

The dichloromethane extracts from *A. dealbata* bark, wood and leaves presented distinct extraction yields. *A. dealbata* leaves showed the highest yield, accounting for 6.2 ± 0.22%, followed by bark extract (2.3 ± 0.25%), while wood presented the lowest yield, accounting for 0.30 ± 0.01%.

The extraction yields obtained for *A. dealbata* bark and wood are in accordance with those already reported in the literature for these morphological parts (2.00 ± 0.06 and 0.36 ± 0.03%, respectively) [15]. Although no data have been reported so far concerning the lipophilic extraction yield of *A. dealbata* leaves, the value obtained is higher than that reported for the hexane extract of *A. sinulata* leaves [23].

### 2.2. Chemical Characterization of the Lipophilic Extract

**Figure 1:**
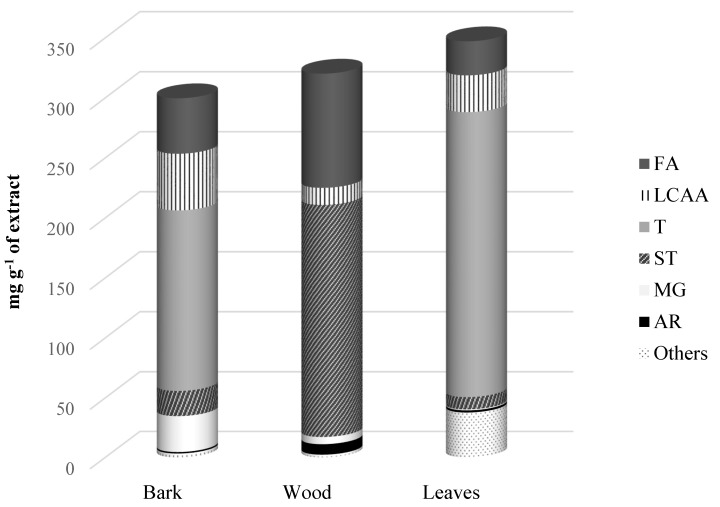
The major families of lipophilic compounds identified in DCM extracts of *A. dealbata* bark, wood and leaves. Abbreviations: FA, fatty acids; LCAA, long-chain aliphatic alcohols; T, terpenic compounds; ST, sterols; MG, monoglycerides and AR, aromatic compounds.

The chemical composition of the dichloromethane extracts of the three morphological parts of *A. dealbata* was studied by GC–MS analysis. Six main families of lipophilic compounds were identified and quantified, namely fatty acids, long-chain aliphatic alcohols (LCAAs), terpenic compounds, sterols, monoglycerides and aromatic compounds (Figure 1). The identification and detailed quantification of the main lipophilic components are summarized in Table 1. The total contents of identified compounds were 976.3 mg kg^−1^ dw in wood, 6872.4 mg kg^−1^ dw in bark, and 21394.5 mg kg^−1^ dw in leaves.

Terpenic compounds were the main family present in the bark and leaves extracts, with contents ranging from 3450.8 mg kg^−1^ dw in bark to 14,635.3 mg kg^−1^ dw in leaves (representing 50.2% and 68.4% of the total content of identified compounds in bark and leaves, respectively). In opposition, terpenic compounds were not found in the wood extract. Actually, the lipophilic extract of this morphological part was shown to be mainly composed of sterols, with a content of 590.4 mg kg^−1^ dw, representing 60.5% of the total content of identified compounds in wood.

#### 2.2.1. Fatty Acids

Fatty acids represent between 8.2% (leaves) and 29.7% (wood) of the total lipophilic components extracted from the different morphological parts of *A. dealbata*. Total saturated fatty acids accounted for 168.7 mg kg^−1^ dw in wood, 946.1 mg kg^−1^ dw in bark and 1415.9 mg kg^−1^ dw in leaves. Saturated fatty acids were found at higher contents than unsaturated ones, accounting for 81.0% and 89.3% of the total fatty acids content in leaves and bark, respectively. The chain length of fatty acids ranged from 12 to 32 carbon atoms. Hexadecanoic acid was the most abundant saturated fatty acid found in leaves and wood, accounting for 389.1 and 69.7 mg kg^−1^ dw, respectively, whereas triacontanoic acid was the most abundant saturated fatty acid in bark, accounting for 188.9 mg kg^−1^ dw.

Considerable amounts of unsaturated fatty acids were found in bark, wood and leaves, with contents ranging from 95.7 to 331.5 mg kg^−1^ dw. These components represented about 40.7% of the total fatty acids detected in wood, accounting 118.0 mg kg^−1^ dw. Octadeca−9,12-dienoic acid, an ω-6 fatty acid, was the most abundant unsaturated fatty acid detected in bark and wood, accounting for, respectively 39.6 and 97.2 mg kg^−1^ dw, while the ω-3 fatty acid octadeca-9,12,15-trienoic acid was the major unsaturated fatty acid observed in leaves, accounting for 116.2 mg kg^−1^ dw.

Unsaturated acids, despite being essential for human health, are not synthesized by the body. These compounds are associated with the prevention and/or treatment of chronic and acute diseases such as cardiovascular disease, cancer, osteoporosis and immune disorders [24]. Their abundance in *A. dealbata* lipophilic extracts thus highlights the potential of this fraction to be exploited in nutraceutical applications.

So far, only a limited number of fatty acids were identified in *A. dealbata*, and particularly in the bark, namely hexadecenoic, octadeca-9,12-dienoic, docosanoic, tetracosanoic, octacosanoic and triacontanoic acids [17]. All the remaining fatty acids were identified here for the first time as bark constituents. In addition, fatty acids identified in wood and leaves are reported here for the first time.

#### 2.2.2. Long-Chain Aliphatic Alcohols

Long-chain aliphatic alcohols (LCAA) were also detected in all extracts and represented a significative fraction of the total content of identified lipophilic compounds. The highest LCAA contents were found in leaves and bark (1891.5 and 1082.5 mg kg^−1^ dw, respectively), while a considerably lower amount was observed in wood (43.7 mg kg^−1^ dw). Triacontan-1-ol was the major LCAA in wood and leaves, with contents of 15.2 mg kg^−1^ dw and 1035.2 mg kg^−1^ dw, respectively. The bark extract showed the highest number of LCAA, presenting chain lengths from 12 to 32 carbon atoms. Hexacosan-1-ol was the most abundant LCAA in this morphological part, reaching up to 265.5 mg kg^−1^ dw. Although no information has been reported so far regarding the LCAA content in the different morphological parts of this tree, it was suggested in a previous study [17] that hexacosan-1-ol was the major LCAA of *A. dealbata* bark DCM extract, which is in accordance with the findings of the present study.

With the exception of tetracosan-1-ol, hexacosan-1-ol, octacosan-1-ol and triacontan-1-ol, which were previously identified in *A. dealbata* bark [17], all the other LCAA (Table 1) are reported here for the first time in this morphological part. Considering wood and leaves fractions, to the best of our knowledge, this is the first study reporting the LCAA profile in these morphological parts of *A. dealbata*.

#### 2.2.3. Terpenic Compounds

**Figure 2:**
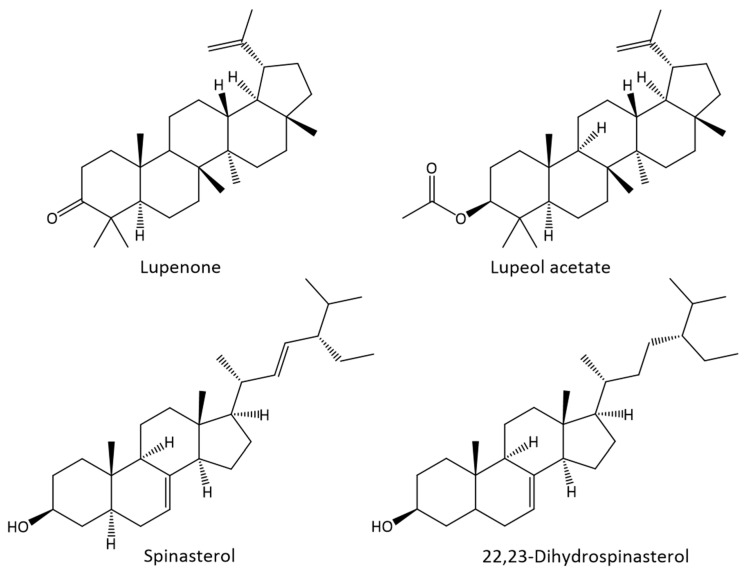
Chemical structures of the major constituents identified in the *A. dealbata* bark, wood and leaves lipophilic extracts.

Terpenic compounds were the main family identified in bark and leaves lipophilic extracts, accounting for about 50.2% and 68.4% of the total content of identified lipophilic compounds, respectively (Figure 1). Terpenic compounds were absent in the wood extract, while two triterpenic compounds were found in the bark lipophilic extract in relatively high amounts, namely lupenone (1293.7 mg kg^−1^ dw) and lupenyl acetate (2157.1 mg kg^−1^ dw) (Figure 2). Interestingly, this component was only present in the bark extract. Seven terpenic compounds were identified in the leaves extract, corresponding to a total content of 14,635.3 mg kg^−1^ dw. Among those, lupenone was the most abundant compound of this family in leaves (6946.8 mg kg^−1^ dw), followed by *α*-amyrin (2578.2 mg kg^−1^ dw) and squalene (1747.6 mg kg^−1^ dw). From the identified terpenic compounds, only lupenone has been previously reported as constituent of *A. dealbata* leaves [8]. 

Plant-derived triterpenic compounds have been associated with a wide variety of biological activities. Lupenone, present in bark and leaves extracts (56.4 and 112.7 mg g^−1^ extract, respectively), has been described as a potential therapeutic agent in inflammation, diabetes, virus infection, cancer and Chagas disease [18]. Lupenyl acetate, identified in the bark extract (94.0 mg g^−1^ extract), has been reported to possess in vitro and in vivo anti-inflammatory activity [25,26], highlighting the potential of *A. dealbata* lipophilic fraction for high value applications.

#### 2.2.4. Sterols

Considerable amounts of sterols were observed in the three morphological parts of *A. dealbata*, with contents ranging from 484.2 mg kg^−1^ dw in bark to 635.7 mg kg^−1^ dw in leaves. Wood extract showed to be particularly rich in sterols (Figure 1), with this family representing 60.5% of the total compounds identified. Two Δ^7^ sterols were identified in bark and wood, namely 22,23-dihydrospinasterol (285.8 and 280.7 and mg kg^−1^ dw, respectively) and spinasterol (198.3 and 288.5 and mg kg^−1^ dw, respectively) (Figure 2). In addition, sitostanol was also identified in wood, accounting for 21.2 mg kg^−1^ dw.

From a qualitative point of view, the sterols identified in bark and wood are in accordance with previous reports [15]. However, some differences can be pointed out from a quantitative perspective. Actually, sterols contents observed for bark and wood (484.2 and 590.4 mg kg^−1^ dw, respectively) were considerably higher than those reported before, following a similar extraction procedure (259.4 and 381 mg kg^−1^ dw in bark and wood, respectively) [15]. These differences may arise from the variability caused by geographic origin or edaphoclimatic conditions. Regarding leaves extract, only 22,23-dihydrospinasterol was detected, however at a considerable high content, namely 635.7 mg kg^−1^ dw. As far as we know, this is the first and only sterol identified in *A. dealbata* leaves.

Spinasterol has been reported to show various biological properties, including anticarcinogenic, anti-inflammatory, antitumor, antiulcerogenic activities, and antinociceptive effects [19,21,27,28,29,30].

#### 2.2.5. Monoglycerides

Specific monoglycerides were found in all morphological parts of *A. dealbata*. Bark extract showed the highest percentage of monoglycerides corresponding to 10.1% of the total content of identified lipophilic compounds, while leaves and wood extracts showed relatively low amounts (0.2% and 1.9% of the total content of identified compounds). Further, 1-monodocasanoin and 1-monotetracosanoin were the major monoglycerides found in the bark, representing 149.5 mg kg^−1^ dw and 538.7 mg kg^−1^ dw, respectively, and 1-monohexadecanoin was the only monoglyceride found in all extracts. With the exception of 1-monotetracosanoin, previously reported as constituent of *A. dealbata* bark [17], all other monoglycerides found were identified here for the first time in the different morphological parts of *A. dealbata*.

#### 2.2.6. Aromatic Compounds

Several aromatic compounds were identified in *A. dealbata* bark, wood and leaves (Table 1), with contents ranging between 26.9 mg kg^−1^ dw in wood and 112.0 mg kg^−1^ dw in leaves. Tyrosol was the dominant aromatic compound observed in leaves extract (48.5 mg kg^−1^ dw), accounting for 43.3% of the total aromatic compounds content. Other abundant aromatic compounds were verified in leaves lipophilic extract, namely vanillic and p-coumaric acids (12.4 and 19.8 mg kg^−1^ dw, respectively). Vanillin was the major aromatic compound (6.8 mg kg^−1^ dw) of wood lipophilic extract while syringic acid was the most abundant aromatic compound observed in the bark lipophilic extract (7.8 mg kg^−1^ dw). To the best of our knowledge, all these aromatic compounds are reported here for the first time as components of *A. dealbata*, with exception of syringic and p-coumaric acids that were already described in the literature as constituents of a mixture of aerial parts (wood, bark, and leaves) of *A. dealbata* [31].

#### 2.2.7. Other Components

Finally, other compounds were also identified in high amounts in bark and leaves lipophilic extracts, such as glycerol (20.2 mg kg^−1^ dw in bark and 339.7 mg kg^−1^ dw in leaves) and α-tocopherol (46.1 mg kg^−1^ dw in bark and 1936.1 mg kg^−1^ dw in leaves).

### 2.3. Cytotoxity Evaluation of A. dealbata Lipophilic Extracts

**Figure 3:**
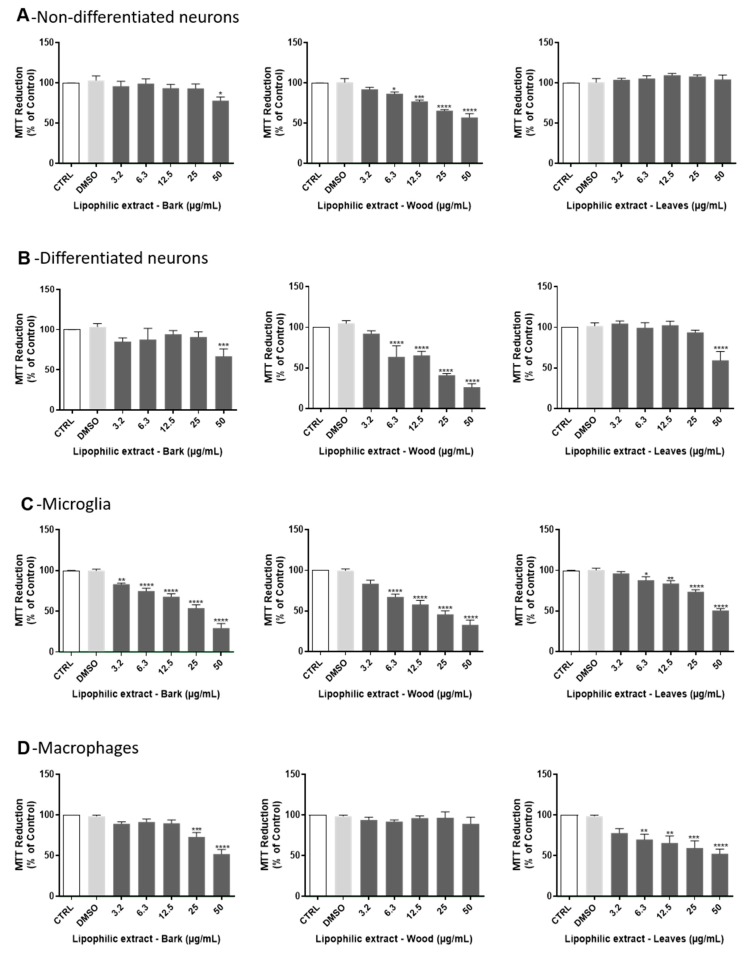

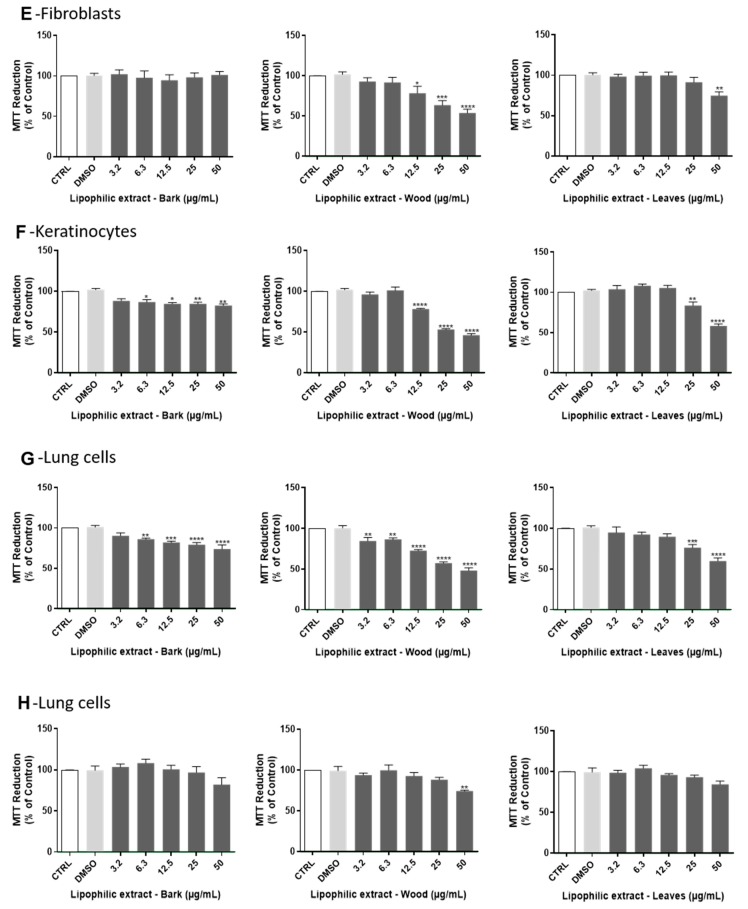
Effect of lipophilic extracts from *A. dealbata* on non-differentiated (**A**) and differentiated (**B**) neuronal N2A, microglia BV-2 (**C**), macrophages Raw 264.7 (**D**), fibroblasts NIH/3T3 (**E**), keratinocytes HaCaT (**F**), lung A549 (**G**) and liver hepatocyte HepG2 (**H**) cells viability. Cells were treated for 24 h at 37 °C with concentrations of 0–50 µg mL^−1^ of lipophilic extracts obtained from bark, wood and leaves of *A. dealbata* and then viability was evaluated by the MTT assay. The results expressed as percentage (%) of control represent the mean ± SEM of at least 3 independent experiments performed in triplicate. Statistical analysis was made by one-way analysis of variance (ANOVA) followed by Dunnett’s multiple comparison test. * *p* < 0.05, ** *p* < 0.01, *** *p* < 0.001, **** *p* < 0.0001 significantly different compared to control.

In order to evaluate the safety of lipophilic extracts from bark, wood and leaves of *A. dealbata*, their cytotoxicity was evaluated by the MTT assay, in cell lines representative of different tissues and organs (brain, innate immune system, skin, lung and liver), namely non-differentiated and differentiated neuronal cells (N2A), microglia (BV−2), macrophages (Raw 264.7), fibroblasts (NIH/3T3), keratinocytes (HaCaT), lung cells (A549), and hepatocytes (HepG2). Regarding non-differentiated neuronal cells (Figure 3 A), after 24 h treatment with the lipophilic extracts, all tested concentrations of leaves extracts were devoid of toxicity, and bark and wood extracts did not exhibit cytotoxicity at doses bellow 50 µg mL^−1^ and 6.3 µg mL^−1^, respectively. In differentiated neuronal cells (Figure 3B), an absence of toxicity was observed after 24 h treatment with the lipophilic extracts at concentrations bellow 50 µg mL^−1^ for leaves and for bark, and 6.3 µg mL^−1^ for wood. In the case of microglia cells, which are the brain-resident immune cells (Figure 3C), non-toxic effects of the lipophilic extracts were observed at 24 h for concentrations bellow 6.3 µg mL^−1^ for leaves and for wood and 3.2 µg mL^−1^ for bark. In macrophages (Figure 3D), which are cells of the peripheral immune system, no significant toxicity was observed for all tested concentrations of wood extracts and non-toxic effects were detected 24 h after cells exposure to the leaves and bark lipophilic extracts at concentrations bellow 6.3 µg mL^−1^ and 25 µg mL^−1^, respectively. Lipophilic extracts of *A. dealbata* were also tested in skin cells: fibroblasts and keratinocytes, representative of the dermis and epidermis, respectively. In fibroblasts (Figure 3E), absence of toxicity was found after 24-h incubation with these extracts at concentrations bellow 50 µg mL^−1^ and 12.5 µg mL^−1^ for leaves and for wood, respectively, while no significant toxicity was observed for bark extract at any tested dose. In the case of keratinocytes (Figure 3F), lipophilic extracts induced significant toxicity in concentrations above 25 µg mL^−1^ for leaves, 6.3 µg mL^−1^ for bark and 12.5 µg mL^−1^ for wood, after 24 h treatment. In lung cells (Figure 3G), non-toxic effects of the lipophilic extracts were observed at 24 h for concentrations bellow 25 µg mL^−1^ for leaves, 6.3 µg mL^−1^ for bark, and 3.2 µg mL^−1^ for wood. Finally, lipophilic extracts were shown to be safe in hepatocytes (Figure 3H), which are the main liver cells. Indeed, after 24 h of incubation, significant toxicity of wood extracts was detected only for concentrations above 50 µg mL^−1^. 

For the first time, a cytotoxicity screening of lipophilic extracts obtained from bark, wood and leaves of *A. dealbata* was performed in several mammalian cell lines representing brain, immune system, skin, lung and liver cells, unveiling its safe doses. The results obtained with the lipophilic extracts from leaves and bark are very promising regarding its future incorporation in oral or topic pharmaceutical formulations, since there is a lack of toxicity in cells of the liver, namely hepatocytes (HepG2), cells of the epidermis, keratinocytes (HaCaT), and the dermis, fibroblasts (NIH/3T3). In fact, Acacia is used in several cosmetic products, which are applied to different parts of the body, however the available data in the literature is considered insufficient to support the safety of the Acacia-derived cosmetics [32]. The absence of leaves extracts’ toxicity towards epithelial alveolar cells supports their potential administration by inhalation. These extracts also displayed low toxicity in non-differentiated and differentiated neuronal cells, and although the pharmacological and medicinal properties of *A. dealbata* have not been studied yet, different species of Acacia are used for the treatment of convulsions and dizziness [33]. Additionally, the wood lipophilic extract did not exhibit cytotoxicity on macrophages for all the concentrations tested, suggesting that these extracts are not deleterious to the innate immune system. These findings encourage the in-depth evaluation of the bioactive effects of *A. dealbata* lipophilic extracts, as well as the signaling pathways and molecular targets modulated by the extracts. In fact, the anti-inflammatory activity of wood extracts from other *Acacia* species have been reported, and is believed to be due to the antioxidant properties, although no direct associations have been made yet [34]. This study highlights the safe bioactive doses of *A. dealbata* lipophilic extracts emphasizing its therapeutic value that should be more extensively investigated for future applications in the pharmaceutical/nutraceutical industry.

## 3. Materials and Methods

### 3.1. Reagents

Dichloromethane (p.a., ≥99% purity) was supplied by Fisher Scientific (Thermo Fisher Scientific, Waltham, Massachusetts, USA). Pyridine (p.a., ≥99.5% purity), N,O-bis(trimethylsilyl)trifluroacetamide (99% purity), trimethylchlorosilane (99% purity), tetracosane (99% purity), hexadecanoic acid (≥99% purity), nonadecan-1-ol (99% purity), syringic acid (99% purity), vanillin (99% purity) and stigmasterol (95% purity) were supplied by Sigma Chemical Co (Madrid, Spain). Betulonic acid (95% purity) was purchased from Chemos GmbH (Regenstauf, Germany).

Dulbecco’s Modified Eagle’s Medium (DMEM), RPMI-1640 Medium, sodium bicarbonate, sodium pyruvate, non-essential amino acid derivatives, L-glutamine, glucose, phenol red, trypsin-EDTA solution, 3-(4,5-dimethylthiazol-2-yl)-2,5-diphenyltetrazolium bromide (MTT), retinoic acid and dimethyl sulfoxide (DMSO) were supplied by Sigma-Aldrich (Lisbon, Portugal). Fetal bovine serum (FBS), penicillin, and streptomycin were purchased from Gibco (Carlsbad, CA, USA).

### 3.2. Sample Collection

Bark, wood, and leaves samples, representative of harvesting biomass residues, were sampled from a 8-year-old *A. dealbata* tree, in September 2018, randomly selected from a property of “The Navigator Company”, Quinta Rei (GPS coordinates 41°12′50″ N, 8°29′34″ W), region of Porto/Valongo, Portugal. Samples were air dried until a constant weight was achieved and grounded in order to select the fraction with a granulometry lower than 1/2 mm prior to extraction.

### 3.3. Characterization of Lipophilic Extracts

#### 3.3.1. Lipophilic Compounds Extraction

Three ground samples (nearly 10 g) of each fraction (bark, wood and leaves) of *A. dealbata* tree were Soxhlet extracted during 8 h with dichloromethane (DCM), a fairly selective solvent to recover lipophilics from biomass [15,35]. The solvent was evaporated to dryness, the extracts were weighed, and the results are expressed as a percentage of dry weight (dw) biomass.

#### 3.3.2. GC–MS Analysis

Previous to GC–MS analysis, 20 mg samples of each dried extract were dissolved in 250 μL of pyridine containing 0.6 mg of tetracosane (internal standard), and then 250 μL of N,O-bis(trimethylsilyl)trifluoroacetamide and 50 μL of trimethylchlorosilane were added and the mixture at 70 °C for 30 min. By adding these last two reagents, the hydroxyl and carboxyl groups of the components of the extracts were converted into trimethylsilyl (TMS) ethers and esters, respectively [15,36].

The derivatized extracts were analyzed by GC–MS using a Trace Gas Chromatograph (2000 series) equipped with a Thermo Scientific DSQ II mass spectrometer (Waltham, Massachusetts, USA). Compounds were separated in a DB-1 J&W capillary column (30 m × 0.32 mm inner diameter, 0.25 μm film thickness, Santa Clara, California, USA), using helium as the carrier gas (35 cm s^−1^). The temperature program was as follows: initial temperature, 80 °C for 5 min; temperature rate, 4 °C min^−1^ up to 260 °C; temperature rate, 2 °C min^−1^ up to 285 °C which was kept for 8 min. The injector and the transfer-line temperatures were, respectively 250 °C and 290 °C, while the split ratio was 1:33. The mass spectrometer was operated in the electron impact mode with energy of 70 eV, and the data were collected at a rate of 1 scan s^−1^ over a range of *m*/*z* 33–700. The ion source was maintained at 250 °C [37].

Compounds were identified by comparing their mass spectra (MS) with a mass spectral library (Wiley-NIST Mass Spectral Library, 2014), by comparing their MS fragmentation profiles with literature data [15,17,37] and, in some cases confirmed based on the characteristic retention times under the same experimental conditions [36,37,38] and/or by injection of standards.

For semi-quantitative analysis, GC–MS calibration was performed with pure reference standards, representative of the main families of lipophilic compounds present in the extracts (hexadecanoic acid, nonadecan-1-ol, betulonic acid, stigmasterol, syringic acid and vanillin), relative to the internal standard (tetracosane). Response factors were determined by averaging six GC–MS runs. Three derivatized extracts were prepared from each morphological part and injected in duplicate. The results presented correspond to the average of the concordant values obtained with a variation of less than 5% (either between injections of the same aliquots and between triplicate extracts of the same morphological part).

### 3.4. Cytotoxicity Evaluation of Lipophilic Extracts

#### 3.4.1. Cell Culture

The mouse neuroblastoma (N2A, from ATCC CCL−131), human keratinocyte (HaCaT, from CLS, Cell Lines Service, Eppelheim, Germany), mouse fibroblast (NIH/3T3, from ATCC CRL-1658) and human lung carcinoma (A549, from ATCC CCL-185) cell lines were cultured with Dulbecco’s Modified Eagle’s Medium (DMEM) (#D5648), supplemented with 10% (*v*/*v*) heat-inactivated fetal bovine serum (FBS), 1% (*v*/*v*) antibiotic solution (10,000 U mL^−1^ penicillin, 10,000 μg mL^−1^ streptomycin), 3.7 g L^−1^ sodium bicarbonate, and 1 mM sodium pyruvate. The N2A cell line culture medium was additionally supplemented with 1% (*v*/*v*) non-essential amino acids. The mouse leukaemic macrophage cell line (Raw 264.7, from ATCC TIB−71) was cultured in DMEM supplemented with 10% (*v*/*v*) non-inactivated FBS, 1% (*v*/*v*) antibiotic solution, 1.5 g L^−1^ sodium bicarbonate and 1 mM sodium pyruvate. The human hepatocellular carcinoma cell line (HepG2, from ATCC HB-8065) was cultured with DMEM (#D5030), supplemented with 10% (*v*/*v*) heat-inactivated FBS, 1% (*v*/*v*) antibiotic solution, 1.5 g L^−1^ sodium bicarbonate, 1 mM sodium pyruvate, 4 mM L-glutamine, 1 g L^−1^ glucose and phenol red. The mouse microglia cell line (BV-2, from ICLC ATL03001, Interlab Cell Line Collection) was cultured in RPMI-1640 medium (#R4130) supplemented with 10% (*v*/*v*) heat-inactivated FBS, 1% (*v*/*v*) antibiotic solution and 2 g L^−1^ sodium bicarbonate. Cells were cultured in 75 cm^2^ flasks and maintained in a humidified 5% CO_2_–95% air atmosphere at 37 °C, and the medium was changed every 2–3 days. Cultures were passaged by trypsinization with Trypsin-EDTA solution 1x when cells reached 70–80% confluence and were sub-cultured over a maximum of ten passages. Raw 264.7 and BV-2 cells were passaged by detaching the cells with a cell scraper. 

#### 3.4.2. Cell Viability Assay

For the evaluation of cell viability, the 3-(4,5-dimethylthiazol-2-yl)-2,5-diphenyltetrazolium bromide (MTT) reduction assay was performed. RAW 264.7, non-differentiated N2A and HepG2, HaCaT, A549, NIH/3T3 and BV-2 cells were seeded in 96-well plates at a density of 9.6 × 10^4^, 2.5 × 10^4^, 2 × 10^4^, 1.6 × 10^4^, 1 × 10^4^ and 8 × 10^3^ cells/well, respectively, and allowed to adhere for 24 h. For the differentiation of neuronal processes, N2A cells were plated in 24-well plates at a density of 2 × 10^4^ cells/well and 24 h later were treated with 10 μM retinoic acid in DMEM with 1% (*v*/*v*) FBS, during 48 h, with medium refreshed every 24 h. Stock solutions of lipophilic extracts from bark, wood, and leaves of *A. dealbata* were prepared in DMSO and stored at −20 °C. On the day of experiment, culture medium was replaced by freshly prepared exposure media [DMEM supplemented with 1% (*v*/*v*) FBS or DMEM with 10 μM retinoic acid and 1% (*v*/*v*) FBS in the case of differentiated N2A cells and RPMI-1640 medium supplemented with 2% (*v*/*v*) FBS in the case of BV-2 cells]. Each plate also included a solvent control [0.2% (*v*/*v*) DMSO prepared in exposure medium]. Dose-response curves were obtained incubating the cells with 0–50 µg mL^−1^ lipophilic extracts from bark, wood and leaves of *A. dealbata* for 24 h at 37 °C. After the incubation period, the medium was removed and a fresh solution of MTT (0.5 mg L^−1^) prepared in Krebs medium (pH 7.4) was added. The different cell lines were incubated with MTT at 37 °C during 30 min (Raw 264. 7 cells), 1 h (HepG2 cells), 2 h (N2A, A549, and HaCaT cells), 3 h (BV-2), or 4 h (NIH/3T3 cells). After incubation, the MTT solution was removed and the formed formazan crystals were dissolved in DMSO. The absorbance was measured at 570 nm in a spectrophotometer (SLT spectra II) after 10 min shaking. The results were expressed as percentage of the absorbance value obtained in control, which was considered 100% and were graphically presented as a percentage of cell viability versus the lipophilic extracts concentration (µg mL^−1^).

#### 3.4.3. Statistical Analysis

The results are presented as the mean ± standard error of the mean (SEM) of the indicated number of experiments. Normality of the data distribution was assessed by the D’Agostino & Pearson and Shapiro-Wilk normality tests. Statistical comparisons between groups were performed by one-way analysis of variance (ANOVA) followed by Dunnett’s multiple comparison test. Significance was accepted at *p* values < 0.05. All statistical calculations were performed using GraphPad Prism software (8.0.2, GraphPad Software Inc., San Diego, CA, USA).

## 4. Conclusions

The present study highlight, for the first time, the detailed chemical composition of the lipophilic fraction of different morphological parts of *A. dealbata,* envisaging their integrated exploitation. The amount and composition of dichloromethane extractives of *A. dealbata* bark, wood and leaves differ significantly. Terpenic compounds represented the major lipophilic family present in bark and leaves extracts, whereas sterols were the dominant components in the wood lipophilic fraction. Leaves extract showed the highest content of lipophilic compounds, with lupenone as the major compound, accounting for 32.5% of the total lipophilic extract. The bark extract was demonstrated to be rich in long-chain aliphatic alcohols and monoglycerides, while the wood extract presented the highest fatty acids content.

All lipophilic extracts of *A. dealbata* exhibited none or low cytotoxicity at the tested doses in different mammalian cell lines representing brain, immune system, skin, lung, and liver cells, highlighting the potential of these fractions to be further exploited for oral or topic pharmaceutical applications.

The present study thus represents a valuable contribution to promote the economic exploitation of this forest by-product through in-depth knowledge of its chemical composition and safety. These findings encourage the evaluation of the bioactivity of non-toxic doses of these lipophilic extracts of *A. dealbata* in order to obtain scientific support for their application in the nutraceutical industry. Additionally, uniformization of procedures to characterize these fractions are an important issue to make possible the comparison between biomass from different geographical origins. Future studies concerning the search for alternative extraction methodologies and solvents, such as supercritical carbon dioxide extraction or extraction with deep eutectic solvents are also crucial to allow the exploitation of this natural resource.

## Figures and Tables

**Table 1:** Chemical composition of dichloromethane extracts from bark, wood and leaves lipophilic of *A. dealbata.*

Rt_(min)_	Compound	mg g^−1^ of Extract			mg kg^−1^ of dw		
		Bark	Wood	Leaves	Bark	Wood	Leaves
	**Fatty acids**	**46.2**	**95.1**	**28.3**	**1060.0**	**290.0**	**1747.4**
	*Saturated fatty acids*	41.2	55.4	23.0	946.1	168.7	1415.9
24.12	Dodecanoic acid	0.1	0.3	0.2	2.9	0.9	12.9
29.26	Tetradecanoic acid	0.1	0.3	0.4	2.7	0.9	24.0
31.65	Pentadecanoic acid	0.1	0.5	n.d.	1.7	1.4	n.d.
33.95	Hexadecanoic acid	5.4	22.9	6.3	124.3	69.7	389.1
36.15	Heptadecanoic acid	0.1	1.1	0.1	3.0	3.3	7.2
38.26	Octadecanoic acid	0.8	8.4	1.0	17.4	25.6	59.4
40.28	Nonadecanoic acid	0.1	0.6	n.d.	1.4	1.7	n.d.
42.25	Eicosanoic acid	0.4	2.3	0.7	8.4	6.9	41.5
44.14	Heneicosanoic acid	0.2	1.6	0.3	3.5	5.0	21.4
45.96	Docosanoic acid	1.6	4.6	0.9	36.5	14.1	55.1
47.73	Tricosanoic acid	0.5	2.8	0.6	12.6	8.5	37.2
49.43	Tetracosanoic acid	6.1	6.2	1.3	141.0	18.8	80.0
51.10	Pentacosanoic acid	0.6	1.7	n.d.	13.9	5.1	n.d.
52.90	Hexacosanoic acid	3.7	2.2	1.4	85.9	6.6	86.3
54.80	Heptacosanoic acid	1.1	n.d.	n.d.	24.5	n.d.	n.d.
56.83	Octacosanoic acid	5.3	n.d.	5.4	120.5	n.d.	334.1
58.87	Nonacosanoic acid	3.1	n.d.	n.d.	72.0	n.d.	n.d.
61.10	Triacontanoic acid	8.2	n.d.	4.3	188.9	n.d.	267.5
65.98	Dotriacontanoic acid	3.7	n.d.	n.d.	84.9	n.d.	n.d.
	*Unsaturated fatty acids*	4.2	38.7	5.4	95.7	118.0	331.5
33.34	Hexadec-9-enoic acid	0.1	0.2	0.1	1.3	0.7	6.1
37.38	Octadeca-9,12-dienoic acid	1.7	31.9	1.7	39.6	97.2	104.4
37.42	Octadeca-9,12,15-trienoic acid	0.4	1.2	1.9	9.4	3.7	116.2
37.58	*cis-*Octadec-9-enoic acid	1.6	4.1	1.5	36.3	12.6	89.6
37.73	*trans-*Octadec-9-enoic acid	0.4	1.3	0.2	9.2	3.8	15.1
	*w-Hydroxyacids*	0.8	1.1	n.d	18.2	3.3	n.d.
52.01	22-Hydroxydocosanoic	0.8	1.1	n.d.	18.2	3.3	n.d.
	**Long-chain aliphatic alcohols**	**47.2**	**14.2**	**30.7**	**1082.5**	**43.7**	**1891.5**
22.02	Dodecan-1-ol	0.01	0.5	0.02	0.3	1.4	1.4
24.73	Tridecan-1-ol	0.1	1.4	0.05	1.2	4.2	3.1
27.35	Tetradecan-1-ol	0.04	2.2	n.d.	0.9	6.8	n.d.
29.85	Pentadecan-1-ol	0.7	2.0	n.d.	15.9	6.2	n.d.
32.19	Hexadecan-1-ol	0.04	0.6	0.1	0.8	1.8	8.1
36.63	Octadecan-1-ol	0.1	0.4	0.1	2.0	1.4	3.1
40.71	Eicosan-1-ol	0.1	n.d.	n.d.	2.0	n.d.	n.d.
44.50	Docosan-1-ol	0.2	0.3	n.d.	4.3	1.2	n.d.
46.30	Tricosan-1-ol	0.2	n.d.	n.d.	3.7	n.d.	n.d.
48.05	Tetracosan-1-ol	3.8	n.d.	n.d.	86.5	n.d.	n.d.
49.73	Pentacosan-1-ol	0.9	n.d.	n.d.	19.8	n.d.	n.d.
51.43	Hexacosan-1-ol	11.6	n.d.	3.0	265.5	n.d.	184.1
53.21	Heptacosan-1-ol	1.5	n.d.	n.d.	34.3	n.d.	n.d.
55.15	Octacosan-1-ol	9.6	1.8	4.2	221.0	5.6	261.0
57.17	Nonacosan-1-ol	2.3	n.d.	n.d.	53.2	n.d.	n.d.
59.25	Triacontan-1-ol	10.8	5.0	16.8	247.9	15.2	1035.2
63.71	Dotricontan-1-ol	5.4	n.d.	6.4	123.3	n.d.	395.6
	**Terpenic compounds**	**150.5**	**n.d.**	**237.4**	**3450.8**	**n.d.**	**14,635.3**
29.00	Neophytadiene	n.d.	n.d.	2.6	n.d.	n.d.	158.8
37.02	Phytol	n.d.	n.d.	5.5	n.d.	n.d.	340.6
48.89	Squalene	n.d.	n.d.	28.4	n.d.	n.d.	1747.6
56.66	*β*-Amyrone	n.d.	n.d.	20.4	n.d.	n.d.	1256.5
57.57	Lupenone	56.4	n.d.	112.7	1293.7	n.d.	6946.8
58.36	*β-*Amyrin	n.d.	n.d.	26.1	n.d.	n.d.	1606.8
58.97	*α*-Amyrin	n.d.	n.d.	41.8	n.d.	n.d.	2578.2
60.52	Lupenyl acetate	94.0	n.d.	n.d.	2157.1	n.d.	n.d.
	**Sterols**	**21.1**	**193.7**	**10.3**	**484.2**	**590.4**	**635.7**
58.39	Spinasterol	8.6	94.6	n.d.	198.3	288.5	n.d.
58.69	Sitostanol	n.d.	7.0	n.d.	n.d.	21.2	n.d.
59.63	22,23-Dihydrospinasterol	12.5	92.1	10.3	285.8	280.7	635.7
	**Monoglycerides**	**30.2**	**6.0**	**0.8**	**691.6**	**18.4**	**49.4**
45.14	1-Monohexadecenoin	n.d.	n.d.	0.3	n.d.	n.d.	16.7
45.36	1-Monohexadecanoin	0.1	2.8	0.5	3.4	8.4	32.7
47.55	2-Monolinolein	n.d.	0.5	n.d.	n.d.	1.6	n.d.
48.13	1-Monolinolein	n.d.	2.8	n.d.	n.d.	8.4	n.d.
55.92	1-Monodocosanoin	6.5	n.d.	n.d.	149.5	n.d.	n.d.
60.06	1-Monotetracosanoin	23.5	n.d.	n.d.	538.7	n.d.	n.d.
	**Aromatic compounds**	**1.3**	**8.8**	**1.8**	**29.0**	**26.9**	**112.0**
	*Aromatic aldehydes*	0.2	5.8	0.1	4.4	17.6	9.1
14.86	4-Hydroxybenzaldehyde	0.1	0.3	0.1	1.3	0.8	7.3
19.75	Vanillin	0.1	2.2	n.d.	2.5	6.8	n.d.
24.42	Syringaldehyde	0.02	1.4	0.03	0.6	4.2	1.8
24.53	2,5-Hydroxybenzaldehyde	n.d.	0.8	n.d.	n.d.	2.5	n.d.
28.02	Coniferaldehyde	n.d.	0.5	n.d.	n.d.	1.5	n.d.
31.89	Sinapaldehyde	n.d.	0.6	n.d.	n.d.	1.8	n.d.
	*Aromatic acids*	0.7	2.1	0.8	15.9	6.4	47.7
10.98	Benzoic acid	n.d.	n.d.	0.1	n.d.	n.d.	7.0
23.24	*p*-Hydroxybenzoic acid	0.1	0.04	0.1	1.3	0.1	8.4
26.85	Vanillic acid	0.2	1.2	0.2	5.6	3.8	12.4
26.97	Homovanillic acid	0.1	0.1	n.d.	1.2	0.3	n.d.
30.18	Syringic acid	0.3	0.7	n.d.	7.8	2.2	n.d.
31.09	*p*-Coumaric acid	n.d.	n.d.	0.3	n.d.	n.d.	19.8
	Other aromatic compounds	0.4	1.0	0.9	8.8	2.9	55.3
16.07	Resorcinol	0.1	n.d.	0.1	2.8	n.d.	6.8
21.75	Tyrosol	0.02	0.1	0.8	0.5	0.3	48.5
23.62	Vanillyl alcohol	0.2	0.4	n.d.	5.5	1.2	n.d.
27.76	*p*-Coumaric alcohol	n.d.	0.5	n.d.	n.d.	1.4	n.d.
	**Others**	**3.2**	**2.3**	**37.7**	**74.4**	**6.9**	**2323.2**
13.44	Glycerol	0.9	1.5	5.5	20.2	4.6	339.7
15.57	*trans*-Erythronoic acid-*γ*-lactone	0.2	0.5	0.4	4.2	1.6	21.6
17.01	*cis*-Erythronoic acid-*γ*-lactone	0.2	0.2	0.4	3.8	0.7	25.7
54.54	*α*-Tocopherol	2.0	n.d.	31.4	46.1	n.d.	1936.1
	*Total*	*299.6*	*320.2*	*347.1*	*6872.4*	*976.3*	*21,394.5*
